# Remote monitoring in older adults with cancer, opportunities and challenges: a narrative review

**DOI:** 10.1007/s40520-025-03161-x

**Published:** 2025-08-20

**Authors:** Evelyne Liuu, Nicolò Matteo Luca Battisti, Angeline Galvin, Sarah Compton, Tania Kalsi, Marc Paccalin, Simon Valero, Pierre Soubeyran, Carly Welch

**Affiliations:** 1 Department of Geriatrics, Poitiers University Hospital, Poitiers, France; 2 Centre d’Investigation Clinique CIC1402, Inserm, University of Poitiers, Poitiers, France; 3 Laboratoire MOVE (EA6314), Faculty of Sport Sciences, University of Poitiers, Poitiers, France; 4 Department of Twin Research & Genetic Epidemiology, King’s College London, 3rd Floor D Block South Wing, St Thomas’ Campus, London, SE1 7EH UK; 5 Royal Marsden Hospital NHS Foundation Trust, London, UK; 6 Bordeaux Population Health Research Center, University of Bordeaux, Epicene team, UMR 1219, Inserm, Bordeaux, France; 7 Department of Ageing and Health, St Thomas’ Hospital, 9th Floor North Wing, Westminster Bridge, London, SE1 7EH UK; 8 Department of Medical Oncology, Bergonie Institute, Comprehensive Cancer Centre, Bordeaux, France; 9 University of Bordeaux, Vinco team, UMR 1218, Inserm, Bordeaux, France

**Keywords:** Remote monitoring, Geriatric oncology, Patient-reported outcome measures, Wearable electronic devices, Patient care planning or continuity of patient care

## Abstract

**Background:**

The ageing population has led to more cancer cases among older adults, who face higher risks of treatment-related adverse events, functional decline, and unplanned healthcare use. Traditional assessments like Eastern Cooperative Oncology Group and Karnofsky Performance Status lack sensitivity for this group, highlighting the need for new methods to monitor symptoms and functional changes in cancer care. This review examines remote monitoring technologies for older adults with cancer, focusing on their potential and challenges.

**Main body:**

E-health tools such as electronic patient-reported outcomes (ePROs) and wearable devices enable continuous monitoring of symptoms, treatment toxicity, functional status, and adherence. Although benefits like fewer hospitalisations and better survival are shown in younger populations, evidence for older adults is limited. Early studies indicate these technologies are feasible and well-received by older patients but face barriers including digital literacy, cognitive and physical impairments, and healthcare system readiness. Devices like activity trackers and smartphones may detect functional decline and fall risk, though optimal intervention criteria remain unclear. Incorporating e-health into geriatric assessment and survivorship care could foster personalized, proactive management.

**Conclusion:**

Remote monitoring technologies hold promise for enhancing symptom and functional assessment in older adults with cancer, supporting age-appropriate care. However, robust geriatric-specific evidence is lacking. Future research should address technological challenges, validate clinical thresholds, and assess long-term outcomes. Integrating these tools within multidisciplinary frameworks can improve care delivery throughout the cancer journey.

**Supplementary Information:**

The online version contains supplementary material available at 10.1007/s40520-025-03161-x.

## Introduction

### Maintaining physical function, one of the main priorities for older adults receiving cancer care

Despite unquestionable progress in cancer prevention and control, cancer diagnosis, morbidity and mortality rates are still on the rise around the world every year. These trends are placing an increasing burden on healthcare systems [1]. The growing incidence of cancer in individuals aged 65 and over is a global challenge, as the World Health Organization (WHO) predicts that the number of people aged 80 and over will triple between 2020 and 2050 [2]. Nearly two-thirds of newly diagnosed cancer patients are now aged 65 and older [3], and by 2030, 70% of cancer survivors will be over this age [4].

During cancer treatment, older patients may experience new emerging health problems or deteriorating comorbidities, alongside symptoms that may be related to cancer progression or cancer treatment. Also, older patients with cancer are at higher risk of adverse events, such as treatment toxicity, early treatment discontinuation, unplanned readmissions, and even early mortality on cancer treatments [5–8]. If not recognised early, these symptoms can quickly become established and worsen, leading to the risk of functional impairments, decline in quality of life (QOL), and premature discontinuation of treatment and unplanned use of healthcare systems. In routine cancer care, the delay of outpatient follow-up can vary from weeks to months. Physician evaluations of patients’ functional performance, such as the Eastern Cooperative Oncology Group (ECOG) Performance Status (PS) and the Karnofsky Performance Status (KPS) are widely used in oncology, but may not be fully reliable in older adults with cancer, as they are not validated in older individuals and not always relevant in older age [9–11]. These scores show a high degree of inter-observer variability and a moderate degree of agreement with patient assessment [12, 13]. They also do not correlate with the risk of adverse events on chemotherapy [14]. These measures of PS are based on a single global assessment during clinics and may consider the patient to be functioning as normally without taking into account the impairments that would be highlighted by a holistic assessment [11]. However, a large proportion of adverse events in older adults, particularly those related to chemotherapy, may be preventable by considering patient-reported in order to guide cancer management with proactive early interventions and broader care coordination [15].

### Patient reported outcomes in geriatric oncology: opportunities and challenges

A PRO (patient-reported outcome) is an outcome reported directly by the patient, without interpretation or analysis of the response by a clinician or anyone else [16]. It relates to the patient’s health, QOL or functional status, regarding his/her healthcare or treatment [17–19]. Recently, an international consensus of experts developed recommendations about PROs in older individuals with cancer and identified health domains that may be of particular interest in this population: functional autonomy, nutritional status, cognition, and mood [20]. In addition, these experts emphasised the importance of assessing patients’ QOL, symptoms, and level of satisfaction with specific self-report tools. Similarly, the European Society for Medical Oncology (ESMO) guidelines state that PRO questionnaires are valid, reliable and responsive to change, that they should be administered although they observed that the evidence on the feasibility of PRO follow-up in patients aged 75 years and older is lacking [21]. Symptoms may occur throughout the treatment trajectory and even upon its completion, at a time when the patient is in convalescence or in complete remission. Traditional care standards have limited the assessment of patients during cancer care to one-time interactions during face-to-face consultations or, in some cases, over the phone or through home services. This limitation of PROs assessment means that physicians receive a limited amount of information in a short period of time that imperfectly reflects the patient’s overall experience. The vicious circle of undiagnosed or late diagnosis of an adverse symptom may lead to unplanned healthcare resource use, placing a heavy burden on oncology professionals and financial expenditure on the healthcare system.

Results from studies in middle-aged patients confirm the effectiveness of telephone monitoring in managing chemotherapy, allowing more dose-intensive drugs to be administered and reducing the incidence of unplanned readmissions [22, 23]. However, a strategy solely relying on nurses supervised by oncologists may be time-consuming, and challenging to implement in the context of the ongoing shortage of trained health professionals (oncologists, geriatricians, nurses, and other healthcare professionals) [24]. To ensure adequate monitoring and care coordination for older adults with cancer, new solutions are needed to identify the patients most in need of care, thereby optimising the use of existing resources.

Remote monitoring via e-health can be a valuable tool for older adults with cancer to identify and manage toxicities. By continuously monitoring patients’ vital signs, symptoms, and treatment adherence, healthcare providers can detect potential problems early and intervene promptly. This can help to improve patient outcomes and quality of life, while also reducing the burden on caregivers. Additionally, remote monitoring can help to reduce healthcare costs by avoiding unnecessary hospitalizations and emergency room visits. Thus, this narrative review explores the interest and feasibility of e-Health in older adults with cancer as well as its opportunities and its challenges.

## Remote monitoring

### e-Health as a new solution for remote monitoring

**Fig. 1:**
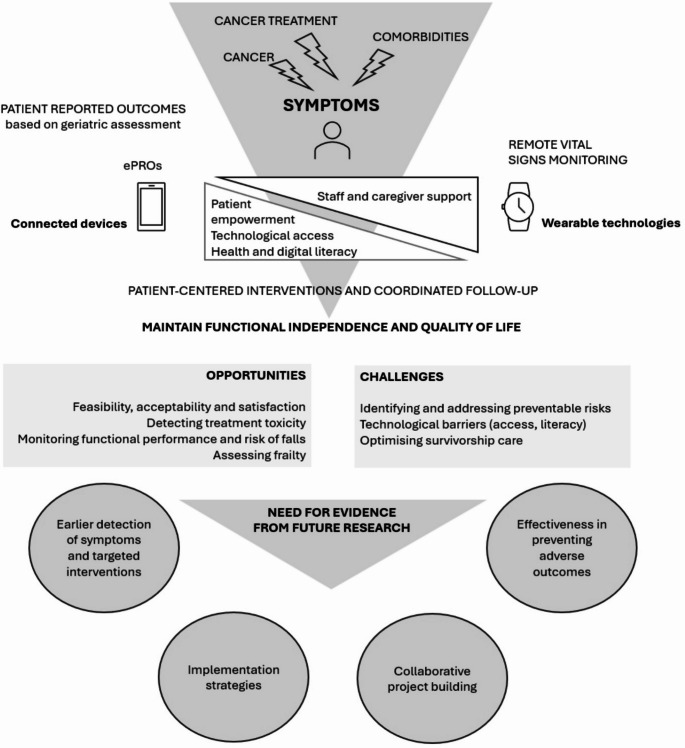
General framework for remote symptom monitoring in older patients with cancer, opportunities, challenges and need of evidence

One potential solution of side effects monitoring and management is e-health, defined as “health services and information delivered or transmitted via the Internet or related technologies”, to bridge the gap between the recommendations of health professionals and health monitoring outside the hospital walls [25]. In recent years, particularly since the COVID-19 pandemic, e-health has developed rapidly and significantly, both in terms of available material and acceptance by patients and healthcare professionals. E-health technologies cover a wide range of possibilities, from tele-consultation through videoconferencing between patient and physician, to wearable technologies that can collect, store and transmit health data. Using e-health, remote symptom monitoring of patients during and after cancer treatment could be based on two complementary technologies, ePRO (electronic patient-reported outcome) and remote vital signs monitoring (Fig. 1) [26].

ePROs are digital-based PROs [15, 27]. Using ePROs questionnaires, patients can report their own symptoms in real time between medical visits. This systematic and frequent reporting can be done using a computer, a mobile phone or other connected devices. This may improve the detection of symptoms and the implementation of early corrective interventions to better anticipate and manage side effects.

Remote monitoring of vital parameters is another solution to assess the patient’s health status with connected devices in a non-intrusive and continuous way. Now owned by two-thirds of adults over the age of 65, smartphones for example give access to multiple integrative communication systems and sensors [28]. They offer possibilities in terms of monitoring of vital signs, health status, and PROs. Other wearable devices exist such as activity trackers, which offer accurate and continuous measurement of physical activity and therefore estimation of mobility [29, 30]. Smartphones and wearable devices could offer new ways of assessing patients’ symptoms and quality of life, particularly by assessing their daily physical functioning [31].

The value of e-health needs to be carefully evaluated, for older adults with cancer, as many questions remain to be answered [32]. In the field of oncology, in younger patients, the use of ePROs improves overall survival (OS) and quality of life during cancer treatment [33]. Benefits of ePROs were also demonstrated in patients with long-term follow-up, as remote symptom monitoring (RSM) using web-based symptom self-assessment also enabled earlier detection of relapse in lung cancer survivors [34].

However, the integration of these technologies in cancer care in older adults remains problematic, partly due to a lack of good quality prospective data on their impact on patient health [21]. Most studies have focused on middle-aged adults, limiting the external validity of the evidence. To date, few studies have investigated RSM in older adults with cancer and concluded that this intervention is feasible, but the available literature is limited and rarely specifically designed for this age group (Additional File 1).

### Remote monitoring implementation in older adults with cancer: feasibility, acceptability and satisfaction

Only few studies were specifically designed for and conducted in adults aged 65 and older with cancer (Table 1).

**Table 1:** Study characteristics

Reference	Country	Nb participants (F/M)	Mean/median age in years (range)	Tumour site	Extension	Cancer treatment type, or survivors	Type of monitoring	NOS quality
Cancel et al. 2024 [35]	France	473 patients (250/223)	79 (Q1-Q3 = 77–82)	various	various	various	ePROs	Low
D’Silva et al. 2018 [53]	Canada	127 patients (73/54)	71.4 ± 9	lung	various	survivors	PROs, PA	Low
Feng et al. 2023 [41]	Canada	90 patients (0/90)	77 (range 65–9)	prostate	metastatic	various	ePROs	Low
Jonker et al. 2023 [52]	The Netherlands	47 patients (16/31)	72.2 ± 5.0 (range 65–85)	various	NR	surgery	ePROs, PA weight and vital signs	Low
Low et al. 2024 [45]	USA	40 patients (20/20)	73.4 ± 4.9	various	NR	survivors	ePROs, PA	Low
Mir et al. 2024 [36]	USA	7 patients (0/7), 3 caregivers	74.9 ± 6.6 (range 67–87)	various	various	various	ePROs, PA, weight	Low
Piau et al. 2019 [37]	France	9 patients (4/5)	83.4 ± 2.1	Haematological malignancies	N/A	chemotherapy	ePROs	Low
Pinto et al. 2021 [64]	USA	20 patients (19/1)	71.6 ± 4.0	various	various	survivors	PA	Low
Soto-Perez-De-Celis et al. 2018 [38]	Mexico	40 patients (23/17)	72.5 (range 65–89)	various	various	chemotherapy	PA	Low

Abbreviations: RCT, randomised clinical trial; PS, prospective study; F, female; M, male; Q1-Q3, first and third interquartile; ePROs, electronic patient-reported outcomes; PA, physical activity; NR, not reported; N/A, not applicable; NOS, Ottawa scale

Studies designed specifically enrolling this population demonstrated acceptable to high rates of feasibility and acceptability in remote monitoring implementation, while the retention rate for active use of devices or ePROs ranged from 66% [35] to 100% [36–38]. However, there is no consensus on the threshold for feasibility and acceptability rates, which makes it challenging to compare these studies’ interventions and results. Basch and colleagues have defined a set of feasibility and acceptability indicators to validate the use of connected tools and ePROs. A threshold of 80% is suggested for the proportion of patients accepting the invitation to participate in a study and of participating patients who complete at least one survey [27]. Several factors may contribute to reduced compliance: physical and cognitive limitations in old age, lack of self-perceived usefulness, lower health and digital literacy, and device complexity in context of technological barriers, including lack of Internet access [39, 40].

In addition to these advantages, user satisfaction has been rated as high in older adults with cancer. Patients indicated that this approach resulted in enhanced motivation and health-promoting behaviours, heightened awareness of symptoms, and more occasions to discuss these symptoms and their global health with healthcare professionals [38, 41]. This high level of satisfaction may reflect patients’ desire to have access to healthcare professionals, although this is not always readily available in practice [42].

## Opportunities for e-Health in older adults with cancer

### Detecting treatment toxicity

No study was specifically designed to validate the efficacy of remote monitoring in older patients (Table 2). However, e-health may provide an opportunity of early detection of symptoms, either by patient self-report or tracker-detected lifestyle change. Increasing evidence suggests that e-health combined with prevention programmes using evidence-based interventions, can lead to improved symptom control, patient quality of life and satisfaction. Studies recruiting younger patients demonstrated reduced hospital admissions and possibly longer overall survival [33, 43]. There is a need for more data on the impact of e-health on early detection of complications in older people receiving cancer treatment, with future studies taking into account the aetiology of symptoms, whether caused by cancer progression or cancer treatment toxicity [3, 44].

**Table 2:** Remote monitoring of symptoms and geriatric domains

	Cancel [35]	D’Silva [53]	Feng [41]	Jonker [52]	Low [45]	Mir [36]	Piau [37]	Pinto [64]	Soto-Perez-De-Celis [38]
**SELF-REPORTED SYMPTOMS**									
General disorders									
Fatigue	RM	RM	RM	RM			RM		
Fever				RM					
Pain	RM	RM	RM	RM					
Gastrointestinal disorders									
Nausea	RM	RM	RM				RM		
Vomiting				RM			RM		
Diarrhea	RM		RM	RM			RM		
Constipation	RM		RM	RM			RM		
Psychological disorders									
Wellbeing	RM	RM	RM	B					
Anxiety	RM	RM	RM						
Depression	RM	RM	RM				RM		
Insomnia	RM	RM	RM						
Nervous system disorders									
Concentration impairment		RM							
Dizziness				RM					
Neuropathy							RM		
Nutritional disorders		B, RM		B, RM		RM	B		
Anorexia	RM	RM	RM	RM			RM		
Respiratory disorders									
Coughing		RM							
Dyspnea	RM	RM	RM	RM					
Skin and subcutaneous disorders									
Skin Rash							RM		
Hair loss		RM							
**GERIATRIC DOMAINS**									
Frailty	B		B	B			B		
Social environment	B	B, RM	B				B	B	B
Functional status		RM	B	B, RM	B		B, RM		B
Physical activity	B	RM		B, RM	B, RM	RM		B, RM	B, RM
Falls					B				
Cognitive status	B						B		B
Incontinence				RM					
Polypharmacy									B
Comorbidities		B	B	B	B				B

Abbreviations: B, baseline; RM, remote monitoring

### Monitoring functional performance and risk of falls

Remote monitoring of physical activity appears to be a potentially feasible and useful strategy for identifying patients at higher risk of functional decline (Table 2) [26]. Some of these studies have shown that real-world data collected by ePROs and related tools can be useful for estimating functional capacity in older cancer survivors and in patients undergoing treatment [45]. Based on a study by Soto-Perez-De-Celis et al., monitoring older patients during chemotherapy for toxicity was feasible and acceptable using a smartphone equipped with an accelerometer [38]. The study showed that a decline in physical activity, with an objective measure of the number of steps taken per day, is common during chemotherapy. This study also documented that patients with low baseline levels of physical activity are at higher risk of adverse events, and that a decline in physical activity is predictive of chemo-induced toxicity. These findings are consistent with the literature showing that reduced steps per day were associated with more severe symptoms [38]. Connected devices may also be used in cancer survivors, over several years. For example, Low and collaborators showed that among older participants who had undergone cancer treatment in the previous five years, data generated from smartphones correlated with measures of physical function, even after adjusting for age and comorbidities. In an exploratory analysis, the number of steps per day from Fitbit^®^ data collected over four weeks was a significant predictor of risk of falls, as a geriatric syndrome [45]. These connected devices may be potentially useful for longitudinal monitoring of physical function [46]. Identifying these patients at risk of functional decline, during survivorship care could help clinicians to suggest corrective interventions and inform shared decision making about treatments to optimise function and quality of life.

Both Low and Soto-Perez-De-Celis teams used modern smartphones equipped with accelerometers, which has several potential advantages over the use of more sensitive devices such as Actigraph^®^ or Fitbit^®^, which are often used in other studies to assess physical activity [38, 45, 47]. Mobile phones are more accessible and can be used both as real-time monitors and direct communication interface between participants and healthcare professionals.

### Assessing frailty and engaging with frail patients

Enabling broader access to vulnerable older adults with cancer may be a key advantage of e-health. On the other hand, although older individuals increasingly use e-health technologies, people with functional impairments have much more limited access. This digital divide may represent an additional care inequality for the most vulnerable individuals [26].

However, integrating connected tools and ePROs may be advantageous in oncology clinical trial design to minimise the burden of clinic visits and study procedures for this specific population [48–50]. The use of smartphone sensors and wearable devices has the potential to provide vital data independent of the patient’s characteristics. By automatically and systematically collecting data over time, these devices can provide physical measurements that are indicators of patients’ symptoms, functional status, and QOL in real time, in their own homes, even in patients who are unable to complete self-report questionnaires.

## Challenges of e-Health in older adults with cancer

### Identifying and addressing preventable risks

Evaluating the modalities of remote monitoring is crucial to guide implementation strategies [51]. The PRO-Common Terminology Criteria for Adverse Events (PRO-CTCAE) [35, 37, 41, 52] and the EORTC QOL questionnaires are the most used PROs scales [53]. The PRO-CTCAE is specifically designed for trials including patients receiving systemic anti-cancer therapy [54]. This set of symptoms is based on the CTCAE, widely used in oncology settings. This adverse event tool allows reference to up to 80 signs common to all types of cancer and chemotherapy. It provides a complementary method for a more comprehensive understanding of the participant’s self-reported symptoms, in line with comprehensive geriatric assessment (CGA) [55].

The choice of symptoms to monitor and vital signs to record must be meaningful to the patient and clinically relevant for the clinician (Table 2) [56]. For example, older adults with cancer frequently report preserving quality of life as a primary concern [57, 58]. However, symptoms may differ in older versus younger adults with cancer in the context of a different burden of comorbidities and polypharmacy [59, 60]. For example, fatigue may be a challenging symptom in old age and may have multifactorial causes whose management is a priority in this population [61].

Identifying the ideal number of symptoms to monitor remotely is important, considering a reasonable balance between a comprehensive clinical monitoring need and the workload this imposes on the patient and the healthcare professional responsible for receiving and processing the alerts [15, 62]. Basch et al. suggested for ePRO programmes with frequent administration (i.e., weekly or daily), that surveys should be short, not exceeding 10 to 15 targeted questions. This length will allow most patients to complete the survey rapidly in less than three minutes, so as not to impose a significant burden. A single survey may be longer, for example up to 30 items to collect broader information on socio-demographic characteristics [27].

In younger subjects, the use of ePROs triggers notifications in a third of cases, and about half of these are generally considered clinically relevant by the case manager [33]. The number of notifications depends on the severity thresholds chosen [13]. Basch et al. suggest that the threshold should be set for a symptom that is considered severe and/or frequent (or a numerical score of 6 out of 10) [27]. The worsening threshold would be set for an increase in symptoms of + 3 out of 10. These thresholds may be arbitrary in older patients, as a small change in symptoms may be considered as significant in older people [13]. Older adults are more prone to experience cancer treatment adverse effects, and even mild toxicity may lead to significant functional decline [26]. The thresholds should also be adjusted according to the expected prevalence of symptoms, considering the characteristics of the cancer, its treatment and patients themself.

This notion of threshold is even more difficult to define when interpreting data from wearable devices. The use of daily step counts was identified as a proxy for patient functional status [63]. Pinto et al. measured step count and physical activity levels using two accelerometers, Fitbit^®^ and Actigraph^®^, and the results were expressed as absolute values of steps per day [64]. No minimum clinically important differences (MCID) were defined a priori. It is the same for Low’s study, in which the number of steps per day was recorded using a Fitbit device [45]. In Soto-Perez-De-Celis’s study, daily steps were recorded using a smartphone and expressed as absolute values and change from baseline [38]. The threshold of 15% decrease in steps over the course of the study was arbitrarily considered significant, and the authors acknowledged that in most cases this decrease was not associated with a significant health event.

The optimal frequency and duration of interventions prompted by remote monitoring should also be considered (Table 3). The questionnaires are occasionally perceived as burdensome, which may potentially dissuade patients over time [65]. Frequency of these interventions should, of course, be based on an estimation of the patient-centred risk of symptoms arising. For example, patients who have undergone cancer surgery may be monitored more closely upon their discharge from hospital, whereas survivors who are undergoing post-treatment surveillance will be monitored at less frequent intervals, although monitoring on a weekly basis was considered optimal in some studies [38, 41]. Also, several ePROs software systems provide patients with the opportunity of self-assessing at any time in case this identifies a problem and warranting contact with their care team. A pragmatic approach may consist of a rapid weekly screening based on a limited number of questions and/or vital signs, combined with telephone follow-up by a nurse focused on the identified risk [27].

**Table 3:** Study design

	Cancel [35]	D’Silva [53]	Feng [41]	Jonker [52]	Low [45]	Mir [36]	Piau [37]	Pinto [64]	Soto-Perez-De-Celis [38]
Study design RCT/PS	PS	PS	PS	PS	PS	PS	PS	PS	PS
Duration	3 months	7 days	3 to 4 weeks	3 months	4 weeks	Mean: 25,3 days	7 weeks	12 weeks	Mean: 18 days
ePROs	Y	N	Y	Y	Y	Y	Y	N	N
Methods of self report	Web-based application	N/A	Web- or phone-based monitoring	Web-based application	Electronic questionnaires	Web-based platform	Smartphone-based chatbot	N/A	N/A
Frequency of report	Weekly	Once	Daily + weekly	Daily	Monthly	Daily	Weekly	N/A	N/A
Sensor-based monitoring	N	Y	N	Y	Y	Y	N	Y	Y
Types of connected devices	N/A	Accelerometer	N/A	Accelerometer and connected devices linked to smartphone or tablet app	Accelerometer and smartphone sensor	Accelerometer and body composition scale	N/A	Accelerometer	Smartphone sensor
Frequency of checks	N/A	Once	N/A	Daily	Daily	Acc: daily, weight: weekly	N/A	Daily	daily
Human resource	NR	NR	Research coordinator + oncology team if needed	Case manager + surgical nurse if needed	physical therapist or cancer rehabilitation nurse	Study team member	Family caregiver in 56%	NR	Research staff member and GP
Collaborative conception	NR	NR	NR	NR	NR	Y	NR	NR	NR

Abbreviations: Y, yes; N, No; RCT, randomized clinical trial; PS, prospective study; ePROS, electronic patient-reported outcomes; Acc, accelerometer; NR, not reported; N/A, not applicable; GP, general practitioner

The total duration of most study interventions does not exceed three months. However, this time frame may be too brief to capture challenges potentially emerging in a typical cancer care pathway. Therefore, a reasonable timeframe for RSM should be a period of 6 to 12 months after the start of a new therapeutic strategy.

### Optimising survivorship care

A person is considered to be a survivor from the time of cancer diagnosis until the end of life [66]. As cancer survival rates continue to improve, more older patients will become cancer survivors. Survivorship is divided into four categories: Acute, which refers to the phase when the patients who require acute interventions at first diagnosis or relapse; Chronic, when the cancer progresses slowly or alternates between periods of remission and relapse; Long-term, when patients are considered to be in clinical remission for long periods of time or for their entire life, but remain at risk of distant relapse or second tumours, and may experience late treatment-related sequelae; Or cured, referring to disease-free individuals whose cancer-specific mortality and life expectancy are equivalent to those of age- and sex-matched members of the general population [67]. Most interventional studies focus on acute survivors (Table 1). In this continuum of care, understanding the essential but unmet needs of older adults will enable better survivorship care planning and anticipate the risk of poor coping and adverse outcomes resulting from the long-term effects of cancer and its treatment, the ageing process and multimorbidity [68, 69]. Experts are calling for the incorporation of geriatric principles into survivorship care planning [70–72]. Scientific groups such as the Cancer and Aging Research Group (CARG) provide resources and guidance on integrating GA during the survivorship phase, including feasible assessments of functional status and health-related quality of life and implementation of targeted interventions [72]. Digital approaches may facilitate more accessible care throughout the entire cancer course, as digital health interventions may enable self-monitoring of symptoms and delivery of care. Recently, an expert panel have developed a core dataset of 19 symptoms related to cancer, cancer treatment, functional decline and the destabilisation of comorbidities for patients during cancer treatment and after its completion [73]. The frequency of reporting of these symptoms and the cut-off for notification have also been determined. Nevertheless, there remains a paucity of evidence to support repeated geriatric screenings and the implementation of interventions led by geriatric assessment in the survivorship phase. This is due to the fact that few studies addressing the effectiveness of such interventions have been specifically designed for older cancer survivors [74, 75].

### Embedding e-Health as part of the existing healthcare strategy

For e-health to be sustainable in clinical practice, all end-users should be as satisfied and motivated as possible. Consideration of ePROs and related tools should be seen as part of a broader strategy of early and personalised interventions, included in CGA [41]. Care coordination with community practitioners and allied health professionals should also be seen as a key element of this strategy. For example, nurse- or pharmacy-led monitoring of patients receiving systemic oral anticancer therapies should also be considered [41].

Participatory development and design may inform implementation based on the preferences and needs of users in the real world [26, 27]. Research into gerontechnology, defined as the use of digital tools designed especially for older people, could get them interested in using these tools. Gerontechnology is an interdisciplinary field that focuses on the user experience and usability for this age group [76, 77]. By encouraging feedback, these tools can be continually improved for the benefit of patients and healthcare professionals [27]. Including caregivers in their development is also key to consider, since, they are frequently involved in symptom reporting for older adults with cancer [37].

This process may be possible only if it involves healthcare professionals that are routinely delivering care to older adults with cancer. However, very few studies include an inclusive multidisciplinary approach in their design (Table 3). Involving and training healthcare professionals to respond effectively to alerts is key and providing them with protected time to carry this out is key. For example, nurses involved in ePROs projects often acknowledge that more time may be required to address an increased number of requests [15]. Importantly, no evidence is available on the time saved by using ePRO and connected tools.

When given a choice, older patients prefer to engage with a single healthcare professional as much as possible, although these interactions may be more time-consuming [15]. Therefore, some patients may prefer telephone monitoring: Wintner et al. reported that in patients invited to complete ePROs at home, one third chose the alternative of telephone calls instead [78]. Basch et al. also investigated users’ perceptions of ePRO use as part of oncology follow-up [33]. Their study found that almost 40% of patients, particularly older adults, used an automated telephone interface rather than a web interface. The evidence suggests that research should allow patients to choose telephone-based and digital-based monitoring. Alternatively, patients may complete ePROs in the clinic. While this option allows ePROs to become widely adopted over time, patients may not be able to report their symptoms in real time at home. This approach does not leverage the advantages of remote self-monitoring. On the other hand, user training and support may enhance their autonomy in using the tools over time.

Training nurses to help patients complete their ePROs at home may be another important enabler of implementation. A study found that 63% of patients aged 80 years or older required support to complete remote ePROs. However, with this approach such, patients may not be able to complete ePROs every time they experience symptoms and is a time-consuming task for nurses.

Finally, a chatbot or alternative AI language model may be considered [37, 79]. A study by Piau’s et al. evaluated the feasibility of a chatbot installed on smartphone and showed that such systems are reliable in recording potentially serious health problems, adherence to care plan and responses provided without the need to contacting directly the care team [37]. A study in younger patients (mean age 63 years) validated the feasibility of an intervention using surveys via an internet-based or automated telephone system to identify PRO-CTCAE symptoms, and educational materials sent to patients by email to help them self-manage these symptoms [80]. Both approaches may save nursing time, but, in the study by Piau et al., most responses (56%) were entered by the caregivers, which highlights potential concerns on the feasibility of such systems in older adults [37].

### The importance of health and digital literacy

Today, two-thirds of adults over the age of 65 own a smartphone, and wrist-worn physical activity sensors are widespread. However, while 75% of people aged 65 and older claim to use the internet, technological accessibility is not so obvious in this age group [35]. In a study by Cancel et al., more than 45% of the participants approached reported a significant technological barrier. Another study investigating the feasibility of ePRO in older patients with prostate cancer found that 72% had daily access to the internet and to emails [35, 81]. However, only 20% of participants were able to complete the ePRO remotely, whereas none reported difficulties in completing the questionnaires on site.

A recent study identified that older adults with cancer and limited health literacy had higher adjusted prevalence of frailty, impaired physical and mental QOL, and recent hospitalisations [82]. Therefore, a digital divide will translate into disparities in care [83]. The patient circumstances and preferences should be considered [40]. By using a wide range of tools including web-based softwares, smartphone applications, connected devices, automated telephone systems, and email/SMS alert options, e-health can reach nearly all patients. Alternatively, patients may be provided with tablets to complete the ePROs at home [35]. Basch et al. observed a significant improvement in overall survival with the use of ePROs, including in patients with little digital experience [15] and Soto-Perez-De-Celis et al. showed that, with minimal training, older adults with cancer were able to use a smartphone correctly to monitor their physical activity, despite their limited digital technology exposure at baseline [38]. Implementation strategies should include a period of training with long-term support to ensure patient-centred empowerment [84].

### Need for evidence from further research

Despite the growing interest in e-health and the potential for remote symptom monitoring, there are very few interventional studies of older adults with cancer that explicitly address their complex needs (Fig. 1). Future evidence is needed to improve this area of research in geriatric oncology and gerontechnology. These studies should include: 1/ Designing interventions that specifically address the unmet needs of older adults with cancer, with early detection of symptoms and provision of personalised corrective interventions, 2/ Implementation research with a particular focus on technological barriers, 3/ Validating the effectiveness of remote symptom monitoring in preventing adverse outcomes and addressing the needs of older patients to maintain functional independence and quality of life, 4/ Promoting a collaborative and multidisciplinary project building, involving all the stakeholders of e-health to ensure care coordination in later life.

## Conclusions

The use of connected tools in healthcare systems and patient demand for technological innovation are increasing [85]. While there is a widespread consensus on the benefits of e-health, the evidence is not mature to validate specific ePROs approaches and connected devices in routine care for older adults with cancer during treatment, or in the survivorship phase. This highlights the need for more research in the context of the ongoing demographic changes and generational shifts suggesting that older adults will become increasingly digital literates. All stakeholders, including patients and caregivers, healthcare professionals, and industry should increasingly engage in investigating the use of e-health in geriatric oncology. Collaborative development of connected tools will facilitate remote monitoring and enhance the value of both patients and healthcare professionals in this new relationship of trust, maximising and coordinating care in the context of existing limitations with financial and human resources. A strategy enabled by gerontechnology may allow rapid identification and interventions to address adverse events in order to enable healthcare systems to meet patients’ needs through personalised, comprehensive and integrated care based on the CGA model [26].

## Supplementary Information

Below is the link to the electronic supplementary material.

Supplementary Material 1

Supplementary Material 2

## Data Availability

No datasets were generated or analysed during the current study.
