# Supplementary material for: Remote monitoring in older adults with cancer, opportunities and challenges: a narrative review
Source: Aging Clin Exp Res. 2025 Aug 20;37(1):249. doi: 10.1007/s40520-025-03161-x (PMC12367841; doi:10.1007/s40520-025-03161-x)
Supplement: Supplementary file 2 — Supplementary Material 2 [file 40520_2025_3161_MOESM2_ESM.docx]

**Article title: Remote monitoring in older adults with cancer, opportunities and challenges: a narrative review**

Journal name: Aging and Clinical Experimental Research

Authors Names: Evelyne Liuu, Nicolò Matteo Luca Battisti, Angeline Galvin, Sarah Compton, Tania Kalsi, Marc Paccalin, Simon Valero, Pierre Soubeyran, Carly Welch

Corresponding author: Evelyne Liuu,

Affiliations: Department of Geriatrics, Poitiers University Hospital, Poitiers, France; Inserm, Centre d'Investigation Clinique CIC1402, University of Poitiers, Poitiers,  France; Laboratoire MOVE (EA6314), Faculty of Sport Sciences, University of Poitiers, Poitiers, France; Department of Twin Research & Genetic Epidemiology, 3rd Floor D Block South Wing, St Thomas’ Campus, King’s College London, London, United Kingdom SE1 7EH

E-mail address: Evelyne.liuu@univ-poitiers.fr

**Additional file.**

The aim of this review is to present the current state of knowledge based on studies of remote symptom monitoring in patients aged 65 years and older during cancer treatment and follow-up, and to determine whether older people with cancer could benefit from these technologies. This article was designed as a narrative review to provide a broad and integrative overview of the current literature on this topic. A narrative approach was chosen due to the heterogeneity of study designs, patients populations, and interventions found in this emerging field. This methodological approach allowed us to incorporate a wide range of evidence sources and expert perspectives to inform future research and practice. Our objective was also to identify opportunities and challenges in knowledge that should be addressed in future studies.

**Methods**

A comprehensive search for articles was conducted to provide an overview of the current knowledge on existing studies on remote monitoring in older patients suffering from cancers.

The research questions were as follows:

1. What types of remote monitoring interventions were specifically designed and conducted in older adults aged 65 and older with cancer (during cancer treatment and follow-up)?

2. What is the quality of the research studies identified?

To answer these research questions, we defined eligibility criteria for the publications in English or French, using the PICOS criteria: Patients (adults 65 years and over with cancer), Intervention (remote monitoring), Comparator (if applicable, with vs. without remote monitoring), Outcomes (feasibility, acceptability, potential impact on clinical outcomes such as overall survival, rate of unplanned hospitalisations, quality of life, detection of symptoms and adverse effects, identification of geriatric frailty and functional impairment), and Study design (clinical trials, prospective or retrospective studies, preliminary studies). If these criteria were not met, publications were excluded. Articles and summaries were also identified by searching the reference lists of selected articles.

Publications for this narrative review were obtained using the PubMed database. The following groups of search terms were used:

1. “remote” AND “monitoring” as text words,

2. “wearable electronic devices” as Mesh terms

3. “patient-reported outcomes” as text words.

We did not include filters on the type of article to avoid excluding relevant publications, but rather screened all the results of our algorithms. Age filter was selected for 65+.

Publications that focused on phone monitoring by healthcare professionals were not included in this review, as considered as the actual standard of care. The data collection, analysis, and interpretation of the included publications were conducted by a single reviewer (EL). The search was completed on September 17th, 2024. Current level of evidence was analysed regarding the NOS criteria (Newcastle–Ottawa quality assessment scale, Stang, Eur J Epidemiol 2010).

**Results**

With our three PubMed algorithms, we screened 187 articles for “remote monitoring”, 86 for “wearable electronic devices” and 1672 for “patient-reported outcomes”. Among these publications, only 9 studies evaluated remote monitoring as a clinical intervention in adults with cancer aged 65 and older: 7 from the first algorithm, 2 from the second (including one redundant), and five from the third one (including 4 redundant from the two first screenings).

1. What types of remote monitoring interventions were specifically designed and conducted in adults aged 65 and older with cancer (during cancer treatment and follow-up)?

In all these nine studies, sample sizes range from 7 to 473 patients (Table 1) [35-38,41,45,52-53,64]. The studies were conducted after 2018 (3 in 2024 and 2 in 2023) in North America (USA, 3; Canada, 2; Mexico, 1) and European settings (France, 2; the Netherlands, 1). The mean or median age ranged from 71.4 to 83.4 y. Most studies included various tumour sites with various stages. Feng only included patients with metastatic prostate cancers, and D’Silva focused on patients with lung cancer [41,53]. The treatment modes were surgery in one study, chemotherapy in two studies, and various in three. Three studies included cancer survivors. At baseline, geriatric assessment were conducted in five studies, with patients considered to be living with frailty ranging from 11% (according to Groningen Frailty Indicator GFI screening tool [52]) to 58% (according to Vulnerable Elders Survey VES-13 [41]) (Table 2). E-health monitoring were ePROs-based in three studies [35,37,41] , physical activity tracking in two [38,64] , and combined in four of them. Two studies also monitored weight remotely [36,52] as a marker of nutritional status. Jonker also collected vital signs (Table 3). Three studies specifically mentioned the use of a smartphone in the study interventions [38,45,52]. Most were daily interventions (7 studies vs. 2 weekly). Study durations ranged from 18 days to 3 months. All interventions targeted patients’ symptoms and/or daily functioning, using the number of steps recorded by a smartphone or an accelerometer. All symptoms monitored were general and not patient-centred in terms of age, baseline demographic, medical and oncological characteristics (Table 2). No monitoring criteria were specifically chosen according to tumour site, stage of extension, and proposed treatment, mainly because the inclusion criteria allowed for various types of cancer, various stages, and various treatments in most cases. Apart from functional status, no geriatric syndrome was specifically monitored over time.

When it was mentioned in the article (4 out of 9), a research team member provided the education about ePROs and connected devices (Table 3). Only two studies included within their methods the way to address any alert from ePROs and online collected data. One advised the patient to contact his/her nurse and/or general practitioner [52], the second one referred to a monitoring nurse, leading to the implementation of specific interventions, such as medication advice, referrals to clinics, and admissions to the emergency room [41].

All studies were prospective preliminary studies, assessing feasibility and acceptability of the intervention (Table 3). Soto-Perez-De-Celis et al reported as main result a feasibility rate of 93% (considering the a priori threshold of ≥ 75% of subjects having used the connected tool for ≥75% of the total duration of the chemotherapy cycle) and an acceptability rate of 85% (for a previously defined threshold of ≥70% of subjects declaring that the connected tool was easy to use) [38]. In another study, the rates of refusal to participate, dropout during the study and non-response were 26%, 4% and 6% respectively [41]. In a postoperative study, dropout and non-response rates using validated scales (usability (SUS) and acceptability (NPS) scales) were estimated at 21% and 10%, which demonstrated the feasibility, usability, and acceptability according to its authors [52]. Once patients consented to participate, they accepted and adhered to wearing and synchronising the connected device, and the postoperative completion rate was high [52].

Another reported result of remote monitoring was user satisfaction, assessed both for patients and healthcare professionals. The studies selected for our review showed participant satisfaction rates ranging from 57% [35] to 95% [41]. Among the reasons for satisfaction, participants emphasised the improvement in motivation and health-promoting behaviour, the fact that there were more opportunities for discussion between patients and healthcare professionals about symptoms and their global health, symptom awareness, and the contribution to health research [41]. This satisfaction was also high among family caregivers [36] and health professionals [38].

Several major challenges for a broader implementation were reported. Access to technology was a major threat, as Cancel et al identified a technological barrier due to no internet access and a reluctance to use the internet or connected apps in 250 of the 473 participants included [35]. Three studies required smartphone ownership as inclusion criteria (Table 3). Regarding the study design, only one article stated that the design process had been the subject of collaborative construction involving all stakeholders, including patients and health professionals, health researchers, and engineers [36]. Only one trial reported that its intervention was part of a usual care strategy [37].

2. What was the quality of the research studies identified?

All these preliminary studies had a low level of evidence, according to the NOS criteria, due to the small number of inclusions, the lack of representativeness of their participants, and the absence of comparison with a non-intervention group (Table 3). Duration of intervention was short (< six months) and no data of clinical outcomes or follow-up were reported.
